# Incidence rates of hepatocellular carcinoma based on risk stratification in steatotic liver disease for precision medicine: A real-world longitudinal nationwide study

**DOI:** 10.1371/journal.pmed.1004479

**Published:** 2024-10-25

**Authors:** Rongtao Lai, Scott Barnett, Xinrong Zhang, Leslie Yeeman Kam, Ramsey Cheung, Qing Xie, Mindie H. Nguyen

**Affiliations:** 1 Division of Gastroenterology and Hepatology, Stanford University Medical Center, Palo Alto, California, United States of America; 2 Department of Infectious Diseases, Ruijin Hospital, Shanghai Jiaotong University School of Medicine, Shanghai, China; 3 Division of Gastroenterology and Hepatology, Palo Alto Veterans Affairs Medical Center, Palo Alto, California, United States of America; 4 Department of Epidemiology and Population Health, Stanford University, Palo Alto, California, United States of America; University of Calgary, CANADA

## Abstract

**Background:**

Detailed subgroup incidence rates for steatotic liver disease (SLD)-related hepatocellular carcinoma (HCC) are critical to inform practice and public health interventions but remain sparse. We aimed to fill in this gap.

**Methods and findings:**

In a retrospective cohort study of adults with SLD from the United States (US) Merative Marketscan Research Databases (1/2007 to 12/2021), we estimated HCC incidence stratified by sex, age, cirrhosis, diabetes mellitus (DM), and a combination of all these 4 factors. We excluded patients with significant alcohol use and chronic viral hepatitis.

We analyzed data from 741,816 patients with SLD (mean age 51.5 ± 12.8 years, 46% male, 14.7% cirrhosis). During a 2,410,166 person-years (PY) follow-up, 1,740 patients developed HCC. The overall HCC incidence yielded 0.72 per 1,000 PY (95% confidence interval [CI, 0.68, 0.75]). The incidence was higher in males (0.95, 95% CI [0.89, 1.01]) compared to females (0.52, 95% CI [0.48, 0.56]) (*p* < 0.001). For those with cirrhosis, the incidence was significantly higher at 4.29 (95% CI [4.06, 4.51]) compared to those without cirrhosis (0.14, 95% CI [0.13, 0.16]) (*p* < 0.001). Additionally, the incidence was higher in patients with DM (1.19, 95% CI [1.12, 1.26]) compared to those without DM (0.41, 95% CI [0.38, 0.44]) (*p* < 0.001). Chronic kidney disease (CKD) was also associated with a higher HCC incidence of 2.20 (95% CI [2.00, 2.41]) compared to those without CKD (0.58, 95% CI [0.55, 0.62]) (*p* < 0.001). Similarly, individuals with cardiovascular disease (CVD) had a higher HCC incidence of 1.89 (95% CI [1.75, 2.03]) compared to those without CVD (0.51, 95% CI [0.48, 0.54]) (*p* < 0.001). Finally, the incidence of HCC was significantly higher in patients with non-liver cancer (3.90, 95% CI [3.67, 4.12]) compared to those without other cancers (0.29, 95% CI [0.26, 0.31]) (*p* < 0.001). On further stratification, HCC incidence incrementally rose by 10-year age intervals, male sex, cirrhosis, and DM, reaching 19.06 (95% CI [16.10, 22.01]) and 8.44 (95% CI [6.78, 10.10]) in males and females, respectively, but only 0.04 for non-diabetic, noncirrhotic aged <40 years patients in both sexes. The main limitation of this methodology is the potential misclassification of the International Classification of Diseases (ICD) codes inherent in claims database studies.

**Conclusions:**

This nationwide study provided robust granular estimates for SLD-related HCC incidence stratified by several key risk factors. In addition to cirrhosis, future surveillance strategies, prevention, public health initiatives, and future research models should also take into account the impact of sex, age, and DM.

## Abstract 2

Rongtao Lai and team estimate hepatocellular carcinoma incidence stratified by a combination of age, sex, cirrhosis, diabetes, and other comorbidities as derived from data of ~750,000 real-world patients with steatotic liver disease.

## Author summary

**Why was this study done?:**

- Hepatocellular carcinoma (HCC) risk among people with steatotic liver disease (SLD) varies greatly by factors such as age, sex, and comorbidities such as diabetes, cardiovascular disease (CVD), chronic kidney disease (CKD), and liver disease severity.
- There is a lack of precise and detailed subgroup data regarding HCC incidence in people with SLD. This study aims to address this knowledge gap.

**What did the researchers do and find?:**

1. Using real-world population data from US patients with commercial insurance, the current study provides precise HCC incidence rate estimates by such factors to inform precision medicine.
2. The overall incidence rate of HCC in individuals with SLD was 0.72 per 1,000 people-years. Patients aged >70 years with cirrhosis and diabetes had the highest HCC incidence. Those aged <40 years without cirrhosis or diabetes aged <40 years had the lowest HCC incidence.

**What do these findings mean?:**

- These findings indicate that precise subgroup estimates help facilitate surveillance and prevention strategies, enrich the decision-making process for cost-effective healthcare, and provide robust evidence to inform future practice guidelines as well as public health policy.
- The main limitation of the study is that it did not evaluate HCC incidence in uninsured/underinsured populations.

## Introduction

Steatotic liver disease (SLD) is the most common chronic liver disease with increasing prevalence and incidence globally [1–3]. In recent decades, SLD has also become a major cause of hepatocellular carcinoma (HCC). Additionally, survival rates for patients with SLD-related HCC are less favorable compared to those for patients with viral hepatitis-related HCC, largely attributed to lack of routine HCC surveillance [4,5]. Together, these data highlight the urgency for better risk stratification to estimate accurate HCC incidence rates to assist with prevention, HCC surveillance, and better HCC detection strategies. The new definition of SLD is an inclusive diagnosis rather than an exclusive disease diagnosis, and it provides a well-defined description that avoids misclassification. At the same time, the new nomenclature aims to minimize the stigmatization of patients [6,7].

Currently, HCC surveillance is recommended for patients with SLD and cirrhosis but generally not for those without cirrhosis whose risk, as a group as a whole, is below the cost-effectiveness threshold [8]. A recent systematic review and meta-analysis pooled data from 18 studies and reported pooled HCC incidence of 0.03 and 4.62 per 100 person-years for patients with SLD without and with cirrhosis, respectively [9]. However, these pooled results were limited by the heterogeneity of the included data, especially in the analysis of patients with noncirrhotic SLD (I^2^ = 98%) [9]. Yet, nearly 40% of SLD-related HCC was reported to occur in patients without cirrhosis [10,11]. Within the noncirrhotic SLD group, higher incidence has also been reported for some subgroups, though data are sparse and inconsistent, highlighting the need for more precise risk stratification data [12,13].

Therefore, leveraging data from a large national population-based cohort of real-world United States (US) patients with SLD, this study aims to fill this gap by estimating the incidence rates of SLD-related HCC stratified by main risk factors such as sex, age, cirrhosis, diabetes mellitus (DM), cardiovascular disease (CVD), chronic kidney disease (CKD), obesity, and smoking. Also, we carefully ruled out other factors that can contribute to HCC, such as significant alcohol use and chronic viral hepatitis. These detailed and precise population-based estimates of incidence rates of SLD-related HCC will provide epidemiological insights and robust data that can guide actionable strategies for clinical practice and public health intervention for the expanding patient population with SLD.

## Methods

### Study design, data source, and study population

We performed a retrospective cohort study utilizing a large, deidentified US administrative claims Merative Marketscan Research Databases to identify patients with SLD based on a prespecified protocol (**S1 Text**). The database is a large national, geographically diverse administrative claims database with private health insurance and Medicare, encompassing over 250 million participants across the US, housing data on inpatient and outpatient diagnoses, procedures, prescription and dispensing history, participant demographics, and enrollment [14,15]. It also covers physician consultations, health plans, and provider types. Additional functions of this database have been described by our previous studies [16,17]. Definition classification was based on the International Classification of Diseases, Ninth and Tenth Clinical Revisions (ICD-9/10-CM) diagnosis codes. We have zero missing data given this coding scheme. Data were extracted from the time period between January 2007 and December 2021. This study is reported as per the Strengthening the Reporting of Observational Studies in Epidemiology (STROBE) guideline (**S1 Strobe Checklist**).

We selected 975,873 adult patients (≥18 years) with at least 1 inpatient or 2 outpatient diagnoses of SLD. Specifically, 2 outpatient codes within 6 months were chosen to minimize misclassification potential. Other forms of liver diseases (viral hepatitis, alcohol-associated liver disease, autoimmune hepatitis, etc.) were excluded to ensure data precision and avoid potential biases [6,7,18]. We also excluded significant alcohol use based on the ICD-9/10-CM diagnosis codes including alcohol use disorder, alcohol dependence, and alcohol use-induced disorders and complications such as alcoholic pancreatitis and alcohol withdrawal (**S1 Table**). To exclude prevalent HCC including those who may have escaped detection at baseline, patients who developed HCC before or at baseline or within 6 months of the SLD diagnosis were excluded. At the end of enrollment, 67,836 cases with less than 12 months of follow-up were censored (**S1 Fig**).

### Ethics statements

The study was approved by the Institutional Review Board at Stanford University, Stanford, California, USA (Approval Number: 13927). Informed consent was waived for the chart review study by the Stanford Research Compliance Office.

### Study endpoints and study definition

The study endpoint was development of HCC as defined by the ICD-9/10-CM diagnosis codes in any diagnostic positions (**S1 Table**). The index/baseline date was the first date of an SLD diagnosis for patients in the no cirrhosis group (i.e., who did not have cirrhosis at any time during the observation period, while the index/baseline date was the date of the first cirrhosis diagnosis for patients with SLD in the cirrhosis group). Compensated cirrhosis (with a cirrhosis code but no code indicating occurrences of decompensation) and decompensated cirrhosis (included current or past occurrences of any of cirrhosis-associated complications such as ascites, spontaneous bacterial peritonitis, variceal bleeding, hepatic encephalopathy, and/or hepatorenal syndrome) were identified using ICD-9/10 diagnosis codes (**S1 Table**). Other variables, including CVD, CKD, obesity, smoking, or non-hepatocellular carcinoma, were also characterized by ICD-9/10 diagnostic codes (**S1 Table**). Patients’ comorbidity burden was assessed using the Charlson Comorbidity Index (CCI) [19].

### Statistics

We presented continuous data as mean ± standard deviation (SD) for continuous variables and categorical data as percentages (%). We assessed non-normally distributed continuous data with the Wilcoxon rank-sum test. Categorical variables were evaluated using the Chi-square test. Continuous variables (age and CCI) were presented as mean ± SD and analyzed using the Wilcoxon rank-sum test. Categorical variables, including care provider types, DM, CKD, CVD, obesity, smoking, and non-liver cancer, were presented as percentages (%) and analyzed using the Chi-square test.

The cumulative incidence function (CIF) was used to calculate the cumulative incidence of HCC. Cumulative incidence and 95% confidence intervals (CIs) are provided. Person-time was calculated, in years, as time from nonalcoholic fatty liver disease (NAFLD) diagnosis to date of HCC or right-censored in December 2021. Gray’s Test for equality of CIF was used to compare groups and subgroup CIFs. In addition to the overall and subgroup analyses by individual risk factors and comorbidities (sex, age, cirrhosis, DM, obesity, smoking, CVD, CKD, and non-liver cancers), we also stratified the cohort by a combination of sex, age groups (<40, 40–49, 50–59, 60–69, ≥70), cirrhosis, and DM to provide detailed subgroup data for HCC risk stratification. We also performed a sensitivity analysis to start study follow-up at 6 months after the study index/baseline date.

Statistical significance was defined by a 2-tailed *P* value <0.05, and all statistical analyses were performed using R (3.5.0) (http://www.r-project.org/) and SAS software version 9.4 (SAS Institute, Cary, North Carolina, USA).

## Results

### Study patients

A total of 975,873 adult patients with SLD were initially identified between January 2007 and December 2021 (**S1 Fig**). After applying our study exclusion criteria, 741,816 patients remained eligible and were included in the study analysis: 108,862 (14.7%) with cirrhosis and 632,954 (85.3%) without cirrhosis.

The mean age of the total study cohort was 51.5 ± 12.8 years and 46% were male (*p* < 0.001) (**S2 Table**). Compared to patients without cirrhosis, those with cirrhosis were older (55.9 versus 50.7 years, *p* < 0.001), had more comorbidities with higher CCI (3.9 versus 2.3, *p* < 0.001), and were more likely to receive care from gastroenterology/hepatology specialists (42% versus 15.9%, *p* < 0.001). The groups without cirrhosis had more obese patients than the group with cirrhosis (62.4% versus 33.9%, *p* = 0.001). There were more smokers in the cirrhotic group (13.5% versus 14.7%, *p* < 0.001). Interestingly, we listed the 10 most common cancers in the non-liver cancer group, and we found that these cancers were also more common in patients with cirrhosis (all *p* < 0.001, **S2 Table**).

### HCC incidence rates

#### Overall HCC incidence rates and risk stratification by individual major risk factors

During 2,410,166 person-years (PY) of follow-up, a total of 1,740 patients developed HCC, yielding an overall HCC incidence of 0.72 per 1,000 PY (95% CI [0.68, 0.75]) (**Table 1**). A significant disparity in HCC incidence was observed across various demographics and comorbidities. The incidence in older individuals was 1.20 (95% CI [1.14, 1.26]), significantly higher than that in younger individuals, which was 0.16 (95% CI [0.14, 0.19]). Similarly, males presented a higher incidence of 0.16 (95% CI [0.14, 0.19]) compared to females with 0.52 (95% CI [0.48, 0.56]). Notably, individuals with cirrhosis, DM, CVD, or CKD presented significantly higher incidences. For individuals with cirrhosis, the incidence was as high as 4.29 (95% CI [4.06, 4.51]), while for those with DM, it was 1.19 (95% CI [1.12, 1.26]) (**Table 1**). The results of overall and subgroup HCC incidence rates remained consistent in sensitivity analysis where follow-up started 6 months after index date (**S3 Table**).

**Table 1:** Hepatocellular carcinoma incidence rates in patients with steatotic liver disease, overall and in subgroups by age, sex, cirrhosis, diabetes mellitus, or other non-hepatic events.

HCC incidence rates	Patient number	Incident HCC/PY	Incidence rate per 1,000 PY (95% CI)	*p* Value
Overall	741,816	1,740/2,410,166	0.72 (0.68, 0.75)	
Age (years)				
<50	305,816	184/1,117,818	0.16 (0.14, 0.19)	<0.001
≥50	436,000	1,546/1,292,349	1.20 (1.14, 1.26)	
Sex				
Female	399,846	681/1,30,4970	0.52 (0.48, 0.56)	<0.001
Male	341,970	1049/1,105,197	0.95 (0.89, 1.01)	
Cirrhosis				
No	632,954	297/2,07,5747	0.14 (0.13, 0.16)	<0.001
Yes	108,862	1,433/334,419	4.29 (4.06, 4.51)	
DM				
No	466,186	594/1,452,768	0.41 (0.38, 0.44)	<0.001
Yes	275,630	1136/957398	1.19 (1.12, 1.26)	
Obesity				
No	467,141	1141/1,417,054.8	0.81 (0.76, 0.85)	<0.001
Yes	274,675	589/993,111.6	0.59 (0.55, 0.64)	
Smoking				
No	640,360	1457/2,067,018.3	0.70 (0.67, 0.74)	<0.001
Yes	101,456	273/343,148.1	0.80 (0.70, 0.89)	
CKD				
No	679,598	1290/2,210,555.5	0.58 (0.55, 0.62)	<0.001
Yes	62,218	440/199,610.9	2.20 (2.00, 2.41)	
CVD				
No	650,451	1055/2,052,768.9	0.51 (0.48, 0.54)	<0.001
Yes	121,365	675/357,397.5	1.89 (1.75, 2.03)	
Non-liver cancer*				
No	648,391	609/2,122,406.0	0.29 (0.26, 0.31)	<0.001
Yes	93,425	1121/287,760.4	3.90 (3.67, 4.12)	

Categorical data were using the Chi-square test. Levels of significance: all *p* < 0.001 between different subgroups.

*Non-liver cancer: Esophageal cancer; Stomach cancer; Colorectal cancer; Pancreatic cancer; Lung cancer; Breast cancer; Cervix uteri cancer; Prostate cancer; Bladder cancer; Kidney cancer; Thyroid cancer; Hematologic cancer.

CI, confidence interval; CKD, chronic kidney disease; CVD, cardiovascular disease; DM, diabetes mellitus; HCC, hepatocellular carcinoma; PY, person-years; SLD; steatotic liver disease.

### Detailed incidence rates stratified by combinations of 3 or more major risk factors

**Fig 1:**
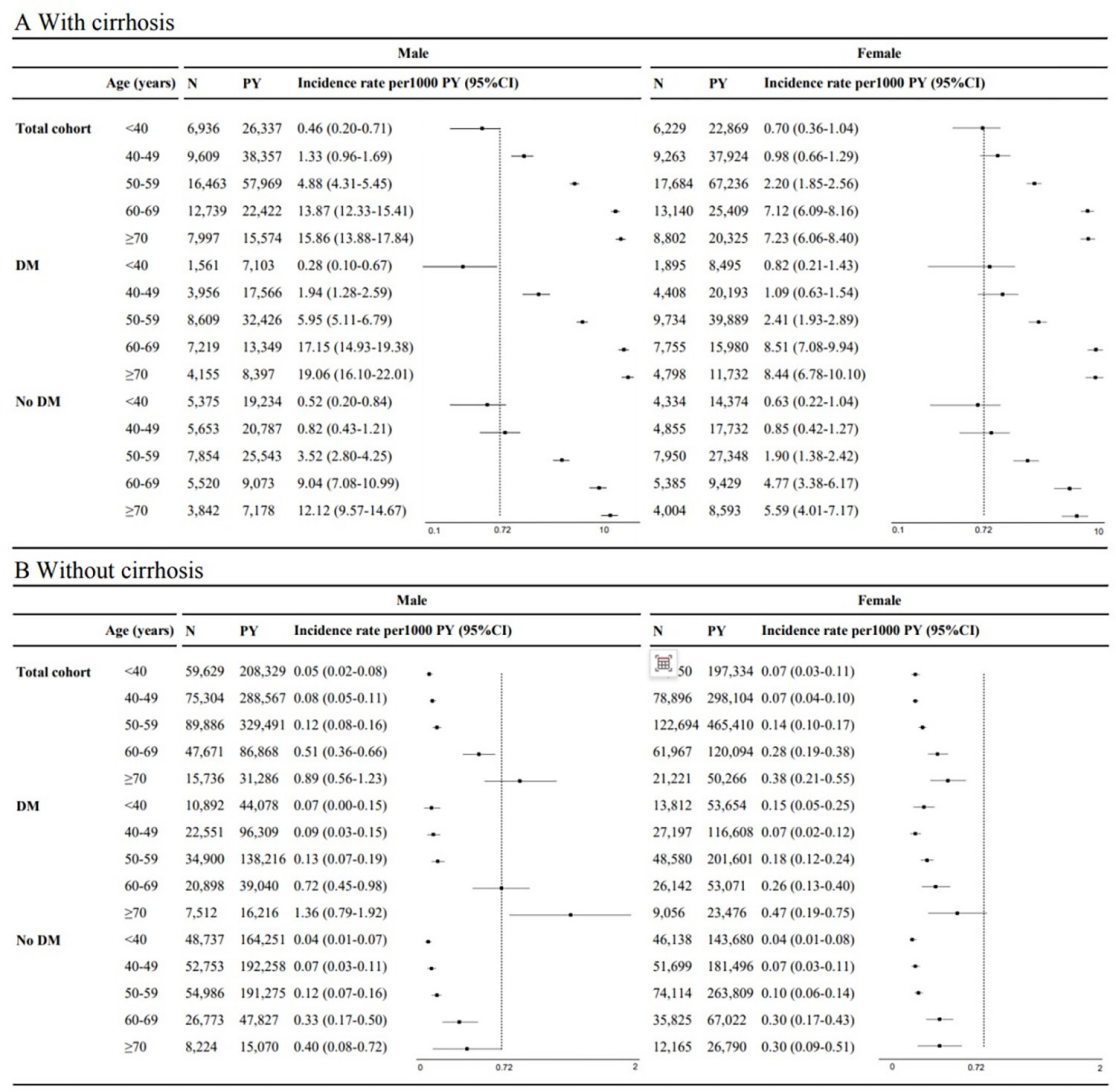
Hepatocellular carcinoma incidence rates per 1,000 PY in subgroups of patients with SLD, stratified by a combination of cirrhosis, sex, age, and DM. Note: The dotted line indicates an overall HCC incidence (per 1,000 PY) of 0.72. CI, confidence interval; DM, diabetes mellitus; HCC, hepatocellular carcinoma; PY, person-years; SLD, steatotic liver disease.

On stratification first by cirrhosis, then sex and age groups in 10-year increments between 40 and 70 years (**Fig 1**), there was a steady rise in HCC incidence with advancing age in both males and females among patients with cirrhosis (**Fig 1A**) as well as among patients without cirrhosis (**Fig 1B**), which further increased among patients with DM upon further stratification. Additionally, regardless of the presence of cirrhosis, HCC incidence was generally higher in males compared to females within most age groups except in the lowest risk group, the noncirrhotic and nondiabetic group. The group with the highest HCC incidence per 1,000 PY were males aged 70 years or older with cirrhosis and DM (19.06, 95% CI [16.10, 22.01]), while the incidence was 8.44 (95% CI [6.78, 10.10]) for the corresponding female subgroup. The groups with the lowest HCC incidence were those younger than 40 years without cirrhosis and without DM (0.04 per 1,000 PY for both males and females). HCC incidence was <2.00 per 1,000 PY for all noncirrhotic subgroups. We further subdivided the patients with SLD into 2 groups, with or without CVD, and then grouped them by sex and age in 10-year increments between the ages of 40 and 70 years. In patients with CVD, HCC incidence was higher than 0.70 per 1,000 PY across all age groups. For patients without CVD, HCC incidence increased significantly, starting at age 50 years. The highest HCC incidence was in males with DM and aged ≥70 years (11.12, 95% CI [8.70, 13.54]) (**S4 Table**). We next stratified by whether or not individuals had non-liver cancer (**S5 Table**), and the incidence of HCC among patients with non-liver cancer between the ages of 40 and 70 years increased steadily with age, in both male and female, especially in the diabetic male aged >70 group (15.57, 95% CI [12.93, 18.20], **S5 Table**).

### Cumulative HCC incidence rates and factors associated with 10-year HCC development

**Fig 2:**
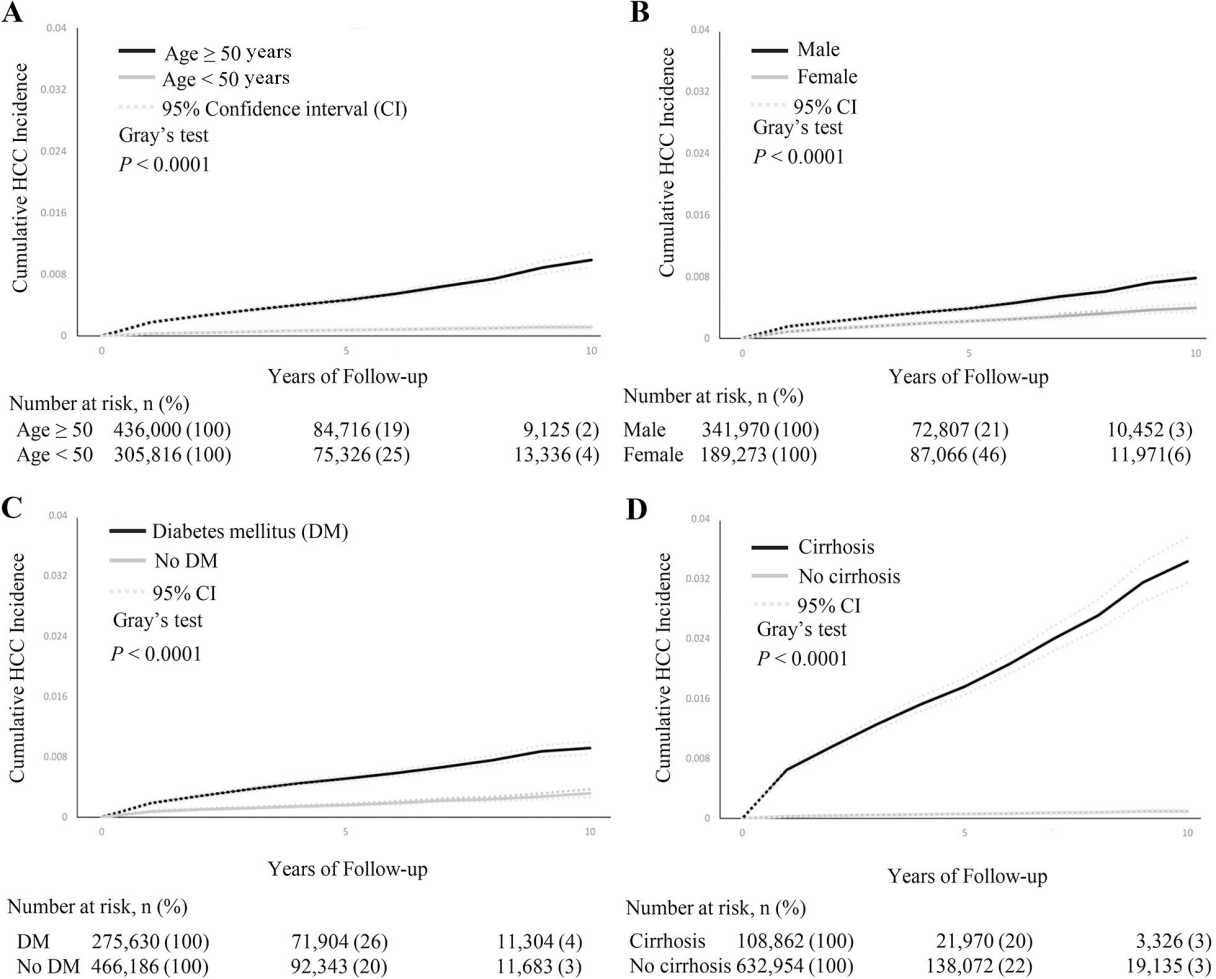
Cumulative hepatocellular carcinoma incidence (%) in subgroups by age, sex, cirrhosis, or DM. Note: (A–D) *P* value for comparison of the 10-year cumulative incidence between subgroups using Gray’s test (all *p* < 0.001). CI, confidence interval; DM, Diabetes mellitus; HCC, hepatocellular carcinoma.

The 10-year cumulative incidence of HCC for patients with SLD varied significantly based on several factors (**Table 2 and Fig 2**). Older age, male sex, DM, smoking, CKD, and CVD were all associated with higher incidences. Among these factors, the presence of non-liver cancers was particularly impactful. Patients with non-liver cancers had a nearly 10-fold higher risk of developing HCC compared to those without. This finding emphasizes the importance of considering the overall cancer history of a patient when assessing their risk of developing HCC (**Table 2**).

Only the cirrhosis group had a 10-year incidence that was greater than 1%, at 3.5% (95% CI [3.2%, 3.9%]), compared to only 0.1% in noncirrhotic patients (*p* < 0.001) (**Table 2 and Fig 2D**). For the subgroup of patients with cirrhosis who were 50 years or older with DM, the 10-year cumulative incidence of HCC was up to 9.7% (95% CI [8.3%, 11.3%]) and 4.1% (95% CI [3.4%, 4.9%]) for males and females, respectively (**S4 Table**). Similar trends of cumulative HCC incidence in subgroups of age, sex, DM, and cirrhosis were observed in sensitivity analysis where follow-up started 6 months after the index date (**S5 Table**).

**Table 2:** Cumulative hepatocellular carcinoma incidence (%) in subgroups by age, sex, cirrhosis, diabetes mellitus, or other non-hepatic events.

Subgroups	5-year cumulative incidence (%, 95% CI)	10-year cumulative incidence (%, 95% CI)	*p* Value*
**Age (years)**			
≥50	0.5 (0.5, 0.5)	1.0 (0.9, 1.1)	<0.001
<50	0.1 (0.1, 0.1)	0.1 (0.1, 0.1)	
**Sex**			
Male	0.4 (0.4, 0.4)	0.8 (0.7, 0.9)	<0.001
Female	0.2 (0.2, 0.2)	0.4 (0.4, 0.5)	
**DM**			
Yes	0.5 (0.5, 0.6)	0.9 (0.9, 1.0)	<0.001
No	0.2 (0.1, 0.2)	0.3 (0.3, 0.4)	
**Cirrhosis**			
Yes	1.8 (1.7, 1.9)	3.5 (3.2, 3.9)	<0.001
No	0.1 (0.1, 0.1)	0.1 (0.1, 0.1)	
**Obesity**			
No	0.3 (0.3, 0.4)	0.6 (0.5, 0.7)	<0.001
Yes	0.3 (0.2, 0.3)	0.6 (0.5, 0.6)	
**Smoking**			
No	0.3 (0.3, 0.3)	0.6 (0.5, 0.6)	0.047
Yes	3 (0.3, 0.4)	0.7 (0.6, 0.8)	
**CKD**			
No	0.2 (0.2, 0.3)	0.4 (0.4, 0.5)	<0.001
Yes	0.9 (0.8, 1.0)	1.9 (1.6, 2.1)	
**CVD**			
No	0.2 (0.2, 0.2)	0.4 (0.3, 0.4)	<0.001
Yes	0.8 (0.7, 0.9)	1.6 (1.4, 1.8)	
Non-liver cancer*			
No	0.1 (0.1, 0.1)	0.3 (0.2, 0.3)	<0.001
Yes	1.6 (1.5, 1.7)	2.9 (2.6, 3.1)	

Cumulative incidences were calculated using the cumulative incidence function, and Gray’s test was employed for statistical analysis.

*p value for comparison of the 10-year cumulative incidence between subgroups. All the subgroups have levels of significance with *p* < 0.001.

*Non-liver cancer: Esophageal cancer; Stomach cancer; Colorectal cancer; Pancreatic cancer; Lung cancer; Breast cancer; Cervix uteri cancer; Prostate cancer; Bladder cancer; Kidney cancer; Thyroid cancer; Hematologic cancer.

CI, confidence interval; CKD, chronic kidney disease; CVD, cardiovascular disease; DM, diabetes mellitus; HCC, hepatocellular carcinoma; PY, person-years.

## Discussion

To our knowledge, this is the largest real-world study evaluating HCC incidence by risk stratification with a national sample of patients with SLD to date. Based on data of close to three-quarter million patients with SLD and over 2.4 million PY of follow-up, the study provided precise and detailed estimates of HCC incidence rates for subgroups stratified not only on key factors such as age, sex, cirrhosis, and DM, but also crucial comorbidities such as CVD, CKD, obesity, and smoking. As such, clinical practice and public health planning should not be based on just the overall HCC incidence as shown to be 0.72 (95% CI [0.68, 0.75]) per 1,000 PY or incidence data based on only 1 risk factor but on a combination of key risk factors such as older age, male sex, cirrhosis, and DM. Though current practice guidelines recommend HCC surveillance in patients with SLD with cirrhosis, structured strategies remain limited. The current study provided detailed incidence rates stratified by background risk to spotlight higher-risk groups, promoting better program surveillance and adherence [8,20,21].

Specifically, detailed stratification revealed significant differences in HCC incidence rates across various demographics and comorbidities. Individuals older than 70 years with both cirrhosis and DM showed the highest risk, with an incidence rate as high as 19.06/1,000 PY. In comparison, younger individuals without cirrhosis and DM presented the lowest risk, with an incidence rate as low as 0.04/1,000 PY. These findings demonstrate the importance of considering multiple risk factors to estimate HCC incidence in patients with SLD. Therefore, the interactions between various risk factors and their impact on HCC development should be investigated in the future to inform targeted prevention and early surveillance strategies. Prior cost-effectiveness studies set the threshold for surveillance of HCC in patients with cirrhosis at an annual incidence of 1.5% [22]. However, recent studies have found a much lower threshold of annual HCC incidence of 0.4% or greater to be cost-effective for semiannual ultrasound and alpha-fetoprotein surveillance among patients with compensated cirrhosis, likely due to improved survival with recent advances in cirrhosis and HCC management. We found that HCC incidence of 0.4% per year or greater to be concentrated in male cirrhotic patients ≥50 years and female cirrhotic patients ≥60 years regardless of DM status.

Furthermore, the current study provided robust data confirming the low risk of HCC among patients without cirrhosis, regardless of age, sex, and presence of DM with HCC incidence of only 1.36 (95% CI [0.79, 1.92]) per 1,000 PY even in the highest risk group (male, aged >70 years, and with DM), thus providing additional support for recommendations against HCC surveillance in SLD without cirrhosis by current practice guidelines [8,20,21]. Our data are in line with pooled results from a recent meta-analysis that included 18 studies, which reported a 0.3 per 1,000 PY HCC incidence in patients with noncirrhotic SLD though the pooled data showed significant heterogeneity [9]. Prior studies have reported HCC incidence ranging from 0.8 to 6.2 per 1,000 PY, with some even noting that up to 50% of HCC cases are found in patients with noncirrhotic SLD [23,24].

Obesity is an independent risk factor for cancer in the general population [25]. However, our study suggests that HCC incidence might be higher in patients with non-obese SLD (0.81, 95% CI [0.76, 0.85]) compared to patients with obese SLD (0.59, 95% CI [0.55, 0.64]). This observation is in line with a prior study with long-term follow-up, which showed a higher risk of HCC in individuals with lean SLD [26]. Additionally, another recent study found that patients with lean SLD might have a worse clinical prognosis, with increased liver-related and overall mortality compared to their obese counterparts [27]. Therefore, future prospective studies with a wider scope to include more practice diversity are necessary to evaluate the risk factors for HCC development in patients with SLD.

For patients who had CVD, HCC incidence was higher across all age groups (>0.71/1,000 PY). In addition, we found that in the non-liver cancer group, the incidence of HCC in patients with SLD with DM increased with age, especially for males over 70 years old, the HCC incidence was as high as 15.57/1,000 PY. Therefore, other comorbidities such as CVD and non-liver cancers may be considered as additional support towards HCC surveillance especially for older males with DM.

It should also be noted that the application of risk-based strategies with cirrhosis is dependent on reasonably accurate diagnosis of cirrhosis, which can be challenging in clinical practice since most patients with SLD do not undergo liver biopsy [28], and even the gold standard liver biopsy can have false negative values [29]. An inherent limitation of such studies is that the diagnosis of cirrhosis often relies on the expertise of the treating physician, which can introduce subjectivity into the results. One of our study limitations is that we could not assess the fibrosis stage because the database is only based on ICD codes and relevant laboratory data to calculate commonly used noninvasive fibrosis tests are missing, such as the FIB-4 index [30]. Thus, judicious and careful assessment of cirrhosis should be broadly based.

The current study has several strengths. First, this study included data from a large nationwide cohort of real-world patients with SLD drawn from both community and referral care centers, making it more generalizable to the general patient population with SLD. Second, the study included observation of close to 2,000 incident HCC over approximately 2.5 million person-years, allowing for detailed granular subgroup analyses combining stratification of major risk factors (age, sex, DM, cirrhosis, CVD, and comorbid non-liver cancer). By providing HCC incidence estimates that surpass prior studies in precision and generalizability, the current study can help identify populations within the population with cirrhotic SLD with heightened HCC risk for enhanced surveillance strategies and adherence, as well as confirming the subthreshold HCC risk among patients with noncirrhotic SLD, even for the higher noncirrhotic risk groups.

The study also has several limitations. Since this is a retrospective study relying on ICD-9/10 codes, it is prone to under coding and misclassification inherent in claims database studies. However, the Merative Marketscan Research Databases is a large and well-established national database with meticulous and rigorous maintenance to help minimize such biases [16]. The database only covered privately insured participants, and data may not be generalizable to the population with government insurance or uninsured population, as those with private insurance tend to have higher income, higher educational levels, better medical follow-up, and possibly lower risk of disease complications [31]. Lastly, the database lacks data that may influence HCC risk such as those of race and ethnicity, lack of information concerning moderate or undiagnosed excess alcohol intake, cases of drug-related SLD, and laboratory data or liver biopsy tests. Additional prospective studies are needed to further stratify HCC risk by race and ethnicity and cirrhosis assessment based on clinical tests.

Our study delineates the incremental increase in HCC risk across advancing 10-year age groups, male sex, presence of cirrhosis, and/or DM to inform precision medicine and to provide foundational population-level data for future modeling studies, public health planning, and intervention for people with SLD. The study identifies the highest risk groups for HCC to be those with cirrhosis, DM, and aged 50 years or older within each sex, while confirming the very low risk among those without cirrhosis regardless of age, sex, and the presence of DM.

## Supporting information

S1 Text Prespecified protocol. (DOC)

S1 Strobe Checklist The Checklist of The Strengthening the Reporting of Observational Studies in Epidemiology (STROBE) Statement. (DOC)

S1 Table ICD-9/10-CM diagnosis codes and procedure codes. (DOC)

S2 Table Baseline demographic characteristics of patients with SLD, overall and by cirrhosis status. (DOC)

S3 Table HCC incidence rates in patients with SLD, overall and in subgroups by age, sex, cirrhosis status, or presence of DM when starting analysis follow-up 6 months after index. (DOC)

S4 Table HCC incidence rates per 1,000 person-years in subgroups of patients with SLD, stratified by a combination of cardiovascular disease, sex, age, and DM. (DOC)

S5 Table HCC incidence rates per 1,000 person-years in subgroups of patients with SLD, stratified by a combination of non-liver cancer, sex, age, and DM. (DOC)

S1 Fig Patient Section from Merative Marketscan Research Databases. Note: *Other liver diseases: Autoimmune hepatitis, primary biliary cholangitis, primary sclerosing cholangitis, hereditary liver disease (alpha-1-antitrypsin deficiency, Wilson’s disease). ** HCC at baseline: any time before or at SLD diagnosis to 6 months after SLD diagnostic date. ICD, International Classification of Diseases; CM, Clinical Modification; SLD, Steatotic liver disease; HCC, hepatocellular carcinoma.
(DOC)

## Abbreviations

CCI: Charlson Comorbidity Index
CI: confidence interval
CIF: cumulative incidence function
CKD: chronic kidney disease
CVD: cardiovascular disease
DM: diabetes mellitus
FIB-4: Fibrosis-4
HCC: hepatocellular carcinoma
HR: hazard ratio
ICD: International Classification of Diseases
IQR: interquartile range
NAFLD: nonalcoholic fatty liver disease
PY: person-years
SD: standard deviation
SLD: steatotic liver disease
