# Supplementary material for: Incidence rates of hepatocellular carcinoma based on risk stratification in steatotic liver disease for precision medicine: A real-world longitudinal nationwide study
Source: PLoS Med. 2024 Oct 25;21(10):e1004479. doi: 10.1371/journal.pmed.1004479 (PMC11548784; doi:10.1371/journal.pmed.1004479)
Supplement: S1 Text — (DOC) [file pmed.1004479.s001.doc]

S1 Text. Pre-specified protocol

Background: Prior studies have suggested that patients with SLD are at higher risk for HCC development regardless of cirrhosis status. However, HCC risk may vary by several factors such as sex, age, race and ethnicity, the presence of cirrhosis and/or diabetes. However, granular HCC incidence data stratified by a combination of relevant background risks are limited for patients with SLD, but they are important to inform patient monitoring, preventive and public health efforts, and future modeling studies. Therefore, our goal was to estimate HCC incidence for a large nationwide cohort of patients diagnosed with SLD in routine clinical practice in the United States with detailed stratification by sex, age, race and ethnicity, cirrhosis status, and the presence of diabetes mellitus (DM).

Methods:

Primary objective:

- Incidence rates of HCC in SLD.
- Incidence rates of HCC were stratified by age, sex, with/without cirrhosis, and diabetes mellitus.

Study design:

This is a retrospective, observational study. Data were obtained from a large deidentified US administrative health care claims Merative Marketscan Research database. Data were extracted from the years 2007-2021.

Diagnosis of SLD and HCC according to the ICD-9/10 CM code.

Criteria

Inclusion Criteria:

- Age ≥18 years.
- Patients with SLD-related cirrhosis diagnosed at any time during the baseline and follow-up periods, the index date was cirrhosis date.
- Patients without SLD-related cirrhosis diagnosis at any time during the baseline and follow-up periods, the index date was SLD date.

Exclusion Criteria

- Presence of any other form of chronic liver disease except SLD.
- Presence of HCC before baseline. Diagnosed with HCC within six months of SLD diagnosis.
- Presence of HCC at baseline.

Statistical analysis
We presented continuous data as mean ± standard deviation (SD) or median (interquartile range [IQR]) for continuous variables and categorical data as percentages (%). We assessed normally distributed continuous data using the Student *t*-test and non-normally distributed continuous data with the Wilcoxon rank-sum test. Categorical variables were assessed using the Chi-square test. The cumulative incidence function (CIF) was used to calculate the cumulative incidence of HCC. Cumulative incidence and 95% confidence intervals are provided. Person-time was calculated, in years, as time from NAFLD diagnosis to date of HCC or right-censored at December 2021. Gray's Test for equality of CIF was used to compare groups and subgroup CIFs. In addition to the overall and subgroup analyses by individual risk factors (sex, age, cirrhosis, and DM), we also stratified the cohort by a combination of sex, age groups (<40, 40-49,50-59, 60-69, ≥70), cirrhosis, and DM to provide detailed subgroup data for HCC risk stratification. We also performed a sensitivity analysis to start study follow-up at 6 months after the study index/baseline date. Statistical significance was defined by a 2-tailed *P* value <0.05, and all statistical analyses were performed using R (3.5.0) (http://www.r-project.org/) and SAS software version 9.4 (SAS Institute, Cary, NC).
