# Supplementary material for: Incidence rates of hepatocellular carcinoma based on risk stratification in steatotic liver disease for precision medicine: A real-world longitudinal nationwide study
Source: PLoS Med. 2024 Oct 25;21(10):e1004479. doi: 10.1371/journal.pmed.1004479 (PMC11548784; doi:10.1371/journal.pmed.1004479)
Supplement: S1 Table — (DOC) [file pmed.1004479.s003.doc]

S1 Table. ICD-9/10-CM diagnosis codes and procedure codes

| Disease | ICD-9/10-CM diagnosis codes |
| --- | --- |
| Steatotic liver disease | 571.8, 571.9; K76.0, K75.81 |
| Hepatocellular carcinoma | 155.0; C22.0, C22.8 |
| Liver cirrhosis | 571.2, 571.5, 572.3, 456.1, 456.21; K70.30, K74.60, K74.69, K76.6, I85.00, I85.10 |
| Hepatic decompensation | |
| Ascites | 789.5, 789.59; R18. 8, K70.31 |
| Spontaneous bacterial peritonitis | 567.23; K65.2 |
| Variceal bleeding | 456.0, 456.2, 456.8; I85.01, I85.11, I86.4 |
| Hepatic encephalopathy | 572.2; K76.82, K72.91, K72.01, K72.11 |
| Hepatorenal syndrome | 572.4; K76.7 |
| Cardiovascular disease | 410-413, 414.0, 427.3, 428, 430-438, 440, 441, 444, V12.5, G45, I20-I22, I48, I50, I60-I66, I70-I79 |
| Chronic kidney disease | 585.1-585.6, 585.9, N18.1-N18.6, N18.9 |
| Obesity | 278.00, 278.01, 278.03, V85.3, E66.0, E66.2, E66.8, E66.9, Z68.3, Z68.4 |
| Smoking | V15.82, 305.1, 649.0, 989.84, Z87.891, F17.200, T65211A, T65212A, T65213A, T65214A, T65221A, T65222A, T65223A |
| Non-liver cancer | 140-239, excluded 155.0, C, excluded C22.0, C22.8 |
| Esophageal cancer | 150, C15 |
| Stomach cancer | 151, C16 |
| Colorectal cancer | 153, 154.0, 154.1, C18-C20 |
| Pancreatic cancer | 157, C25 |
| Lung cancer | 162, C34 |
| Breast cancer | 174, C50 |
| Cervix uteri cancer | 180, C53 |
| Prostate cancer | 185, C61 |
| Bladder cancer | 188, C67 |
| Kidney cancer | 189.0, C64.9 |
| Thyroid cancer | 193, C73 |
| Hematologic cancer | 200-208, C81-96 |
| Hepatitis B virus | 070.20-070.23, 070.30-070.33, V02.61; B16. 0, B16.1, B16. 9; B18. 0, B18.1; B19.1, B19.10, B19.11 |
| Hepatitis C virus | 070.41, 070.44, 070.51, 070.54, 070.70, 070.71, V02.62; B17.10, B18.2, B19.2, B19.20, B19.21 |
| Significant alcohol use | 291, 303.00, 305.00, 357.5, 425.5, 535.30, 535.31, V113, F10.10, G31.2, G72.1, I42.6, K29.20, K29.21, K85.2, K86.0, Z71.4, Z71.41, G62.1, 79.03, 97.73, 98.01, 98.02, 98.03, 98.08, 98.09, 57.10, 57.12, 57.13, E24.4, E86.00, E94.73, K70, K703.1, O35.4, O99.31, R78.0T51, T51, V79.1, Y90, Z658 |
| Other hepatobiliary diseases | 273.4, 275.0, 275.01, 275.1, 570, 571.40, 571.41, 571.42, 571.49, 571.6, 572.0, 572.1, 573.0-573.4, 573.8, 576.1, V02.69; B17.8, B17.9, B18.8, B18.9, E83.00, E83.01, E83.110, E88.01, K71.X, K74.3, K74.4, K74.5, K75.0, K75.1, K75.9, K76.1, K76.2, K76.3, K83.0, K83.01, K83.09, K73.0, K73.1, K73.2, K73.9 |

Abbreviation: ICD-9/10-CM, International Classification of Diseases, Ninth/Tenth Revision, Clinical Modification.
