# Supplementary material for: Incidence rates of hepatocellular carcinoma based on risk stratification in steatotic liver disease for precision medicine: A real-world longitudinal nationwide study
Source: PLoS Med. 2024 Oct 25;21(10):e1004479. doi: 10.1371/journal.pmed.1004479 (PMC11548784; doi:10.1371/journal.pmed.1004479)
Supplement: S2 Table — (DOC) [file pmed.1004479.s004.doc]

S2 Table. Baseline demographic characteristics of patients with SLD, overall and by cirrhosis status

|  | SLD patients, No. (%) | | |  |
| --- | --- | --- | --- | --- |
| Baseline characteristics | Total  N=741,816 | Cirrhosis  N=108,862 | Non-cirrhosis  N=632,954 | *P* value |
| Age, mean (±SD), years | 51.5 ± 12.8 | 55.9 ±13.9 | 50.7 ±12.5 | <0.001 |
| Male | 341,970 (46.1) | 53,744 (49.4) | 288,226 (45.5) | <0.001 |
| Care provider type |  |  |  |  |
| Gastroenterology/hepatology specialist | 146,172 (19.6) | 45,731 (42.0) | 100,441 (15.9) | <0.001 |
| Others* | 269091 (36.3) | 48,910 (44.9) | 220,181 (34.8) | <0.001 |
| CCI, mean (±SD) | 2.5 ± 2.1 | 3.9 ± 3.0 | 2.3 ± 1.8 | <0.001 |
| Diabetes mellitus | 275,630 (37.2) | 54,090 (46.7) | 221,540 (35.0) | <0.001 |
| Chronic kidney disease | 62,218 (16.4) | 18,439 (16.9) | 43,779 (6.9) | <0.001 |
| Cardiovascular disease | 121,365 (16.4) | 29,548 (27.1) | 91,817 (14.5) | <0.001 |
| Obesity | 274,675 (37.0) | 36,391 (33.9) | 237,744 (62.4) | 0.001 |
| Smoking | 101,458 (13.7) | 16,044 (14.7) | 85,414 (13.5) | <0.001 |
| Non-liver cancer | 93,425 (12.6) | 20,027 (18.4) | 73,398 (11.6) | <0.001 |
| Esophageal cancer | 999 (0.1) | 289 (0.3) | 710 (0.1) | <0.001 |
| Stomach cancer | 1,473 (0.2) | 380 (0.4) | 1,093 (0.2) | <0.001 |
| Colorectal cancer | 11,972 (1.6) | 2,678 (2.5) | 9,294 (1.5) | <0.001 |
| Pancreatic cancer | 3,888 (0.5) | 1,061 (1.0) | 2,827 (0.5) | <0.001 |
| Lung cancer | 6,793 (0.9) | 1,461 (1.3) | 5,332 (0.8) | <0.001 |
| Breast cancer | 20,385 (2.8) | 3,556 (3.3) | 16,829 (2.7) | <0.001 |
| Cervix uteri cancer | 4,471 (0.6) | 714 (0.7) | 3,757 (0.6) | 0.014 |
| Prostate cancer | 9,348 (1.3) | 1,727 (1.6) | 7,621 (1.2) | <0.001 |
| Bladder cancer | 1,298 (0.2) | 337 (0.3) | 961 (0.2) | <0.001 |
| Kidney cancer | 6,945 (0.9) | 1,137 (1.0) | 5,808 (0.9) | <0.001 |
| Thyroid cancer | 4,095 (0.6) | 592 0.5) | 3,503 (0.6) | <0.001 |
| Hematologic cancer | 11,330 (1.5) | 3,848 (3.5) | 7,482 (1.2) | <0.001 |

Continuous variables (age and CCI) were presented as mean± standard deviation (SD), and comparisons were made using Wilcoxon rank-sum test; Categorical variables, including care provider type, diabetes mellitus, chronic kidney disease, cardiovascular disease, and non-liver cancer, were presented as percentages (%) and analyzed using Chi-square test. Levels of significance: all P < 0.001 between different subgroups.

Abbreviation: SLD, Steatotic liver disease; SD, Standard deviation; CCI, Charlson comorbidity index.

*Others are defined as all other physician visits.
