# Supplementary material for: Incidence rates of hepatocellular carcinoma based on risk stratification in steatotic liver disease for precision medicine: A real-world longitudinal nationwide study
Source: PLoS Med. 2024 Oct 25;21(10):e1004479. doi: 10.1371/journal.pmed.1004479 (PMC11548784; doi:10.1371/journal.pmed.1004479)
Supplement: S3 Table — (DOC) [file pmed.1004479.s005.doc]

S3 Table. HCC incidence rates in patients with SLD, overall and in subgroups by age, sex, cirrhosis status, presence of DM, or other non-hepatic events when starting analysis follow-up 6 months after index

| HCC incidence rates | Patient number | Incident HCC/PY | Incidence rate per 1,000 PY (95%CI) | *P* value |
| --- | --- | --- | --- | --- |
| Overall | 741,816 | 1,730/2,044,590 | 0.85 (0.81-0.89) |  |
| Age (years) |  |  |  |  |
| < 50 | 305,816 | 184/967,107 | 0.19 (0.16-0.22) | <0.001 |
| ≥ 50 | 436,000 | 1,546/1,077,482 | 1.43 (1.36-1.51) |
| Sex |  |  |  |  |
| Female | 399,846 | 681/1,107,920 | 0.61 (0.57-0.66) | <0.001 |
| Male | 341,970 | 1049/936,669 | 1.12 (1.05-1.19) |
| Cirrhosis |  |  |  |  |
| No | 632,954 | 297/1,763,819 | 0.17 (0.15-0.19) | <0.001 |
| Yes | 108,862 | 1,433/280,771 | 5.10 (4.84-5.37) |
| DM |  |  |  |  |
| No | 466,186 | 594/1,223,026 | 0.49 (0.45-0.52) | <0.001 |
| Yes | 275,630 | 1136/821,564, | 1.38 (1.30-1.46) |
| Obesity |  |  |  |  |
| No | 467,141 | 1141/1,186,841.6 | 0.96 (0.91-1.02) | <0.001 |
| Yes | 274,675 | 589/857,748.2 | 0.69 (0.63-0.74) |
| Smoking |  |  |  |  |
| No | 640,360 | 1457/1,751,440.5 | 0.83 (0.79-0.87) | <0.001 |
| Yes | 101,456 | 273/293,149.3 | 0.93 (0.82-1.04) |
| Chronic kidney disease |  |  |  |  |
| No | 679,598 | 1290/1,875,640.7 | 0.69 (0.65-0.73) | <0.001 |
| Yes | 62,218 | 440/168,949.0 | 2.60 (2.36-2.85) |
| Cardiovascular disease |  |  |  |  |
| No | 620,451 | 1055/1,747,002.5 | 0.60 (0.57-0.64) | <0.001 |
| Yes | 121,365 | 675/297,587.2 | 2.27 (2.10-2.44) |
| Non-liver cancer* |  |  |  |  |
| No | 648,391 | 609/1,802,870.4 | 0.34 (0.31-0.36) | <0.001 |
| Yes | 93,425 | 1121/241,719.3 | 4.64 (4.37-4.91) |

Categorical variables were analyzed using the Chi-square test. Levels of significance: all *p* < 0.0001 between different subgroups.Analysis started follow-up 6 months after the index date. Abbreviation: HCC, Hepatocellular carcinoma; SLD, Steatotic liver disease; DM, Diabetes mellitus; PY, Person-years; CI: Confidence interval

*Non-liver cancer: Esophageal cancer; Stomach cancer; Colorectal cancer; Pancreatic cancer; Lung cancer; Breast cancer; Cervix uteri cancer; Prostate cancer; Bladder cancer; Kidney cancer; Thyroid cancer; Hematologic cancer.
