# Supplementary material for: Incidence rates of hepatocellular carcinoma based on risk stratification in steatotic liver disease for precision medicine: A real-world longitudinal nationwide study
Source: PLoS Med. 2024 Oct 25;21(10):e1004479. doi: 10.1371/journal.pmed.1004479 (PMC11548784; doi:10.1371/journal.pmed.1004479)
Supplement: S4 Table — (DOC) [file pmed.1004479.s006.doc]

S4 Table. HCC incidence rates per 1,000 person-years in subgroups of patients with SLD, stratified by a combination of cardiovascular disease, sex, age, and DM

A Without cardiovascular disease

| Patients |  | Male | | | Female | | |
| --- | --- | --- | --- | --- | --- | --- | --- |
|  | Age (years) | n | PY | Incidence rate per 1000 PY (95%CI) | n | PY | Incidence rate per 1000 PY (95%CI) |
| Total cohort | <40 | 15 | 224,847.5 | 0.07 (0.03-0.10) | 25 | 209,626.5 | 0.12 (0.07-0.17) |
|  | 40-49 | 52 | 295,995.3 | 0.18 (0.13-0.22) | 46 | 303,803.5 | 0.15 (0.11-0.20) |
|  | 50-59 | 209 | 317,682.0 | 0.66 (0.57-0.75) | 151 | 456,722.9 | 0.33 (0.28-0.38) |
|  | 60-69 | 197 | 80,641.8 | 2.44 (2.10-2.78) | 142 | 116,403.6 | 1.22 (1.02-1.42) |
|  | ≥70 | 123 | 16,245.9 | 7.57 (6.23-8.91) | 95 | 30,799.8 | 3.08 (2.46-3.70) |
| DM | <40 | 2 | 47,128.9 | 0.04 (0.00-0.10) | 13 | 57,206.8 | 0.23 (0.10-0.35) |
|  | 40-49 | 27 | 96,842.0 | 0.28 (0.17-0.38) | 22 | 117,804.8 | 0.19 (0.11-0.26) |
|  | 50-59 | 131 | 129,048.2 | 1.02 (0.84-1.19) | 87 | 194,873.7 | 0.45 (0.35-0.54) |
|  | 60-69 | 135 | 35,083.2 | 3.85 (3.20-4.50) | 94 | 50,318.7 | 1.87 (1.49-2.25) |
|  | ≥70 | 81 | 7,284.2 | 11.12 (8.70-13.54) | 56 | 13,195.4 | 4.24 (3.13-5.36) |
| No DM | <40 | 13 | 177,718.6 | 0.07 (0.03-0.11) | 12 | 152,419.7 | 0.08 (0.03-0.12) |
|  | 40-49 | 25 | 199,153.3 | 0.13 (0.08-0.17) | 24 | 185,998.7 | 0.13 (0.08-0.18) |
|  | 50-59 | 78 | 188,633.8 | 0.41 (0.32-0.51) | 64 | 261,849.2 | 0.24 (0.18-0.30) |
|  | 60-69 | 62 | 45,558.6 | 1.36 (1.02-1.70) | 48 | 66,084.9 | 0.73 (0.52-0.93) |
|  | ≥70 | 42 | 8,961.8 | 4.69 (3.27-6.10) | 39 | 17,604.4 | 2.22 (1.52-2.91) |

B With cardiovascular disease

| Patients |  | Male | | | Female | | |
| --- | --- | --- | --- | --- | --- | --- | --- |
|  | Age (years) | n | PY | Incidence rate per 1000 PY (95%CI) | n | PY | Incidence rate per 1000 PY (95%CI) |
| Total cohort | <40 | 7 | 9,818.6 | 0.71 (0.18-1.24) | 5 | 10,575.8 | 0.47 (0.06-0.89) |
|  | 40-49 | 22 | 30,925.6 | 0.71 (0.41-1.01) | 12 | 32,224.8 | 0.37 (0.16-0.58) |
|  | 50-59 | 114 | 69,777.8 | 1.63 (1.33-1.93) | 61 | 75,922.6 | 0.80 (0.60-1.01) |
|  | 60-69 | 158 | 28,647.9 | 5.52 (4.66-6.38) | 73 | 29,099.1 | 2.51 (1.93-3.08) |
|  | ≥70 | 152 | 30,614.2 | 4.97 (4.18-5.75) | 71 | 3,9791.2 | 1.78 (1.37-2.20) |
| DM | <40 | 3 | 4,051.8 | 0.74 (0.00-1.58) | 2 | 4,941.3 | 0.40 (0.00-0.97) |
|  | 40-49 | 16 | 17,033.5 | 0.94 (0.48-1.40) | 8 | 18,995.5 | 0.42 (0.13-0.71) |
|  | 50-59 | 80 | 41,593.9 | 1.92 (1.50-2.34) | 46 | 46,615.7 | 0.99 (0.70-1.27) |
|  | 60-69 | 122 | 17,305.9 | 7.05 (5.80-8.30) | 56 | 18,733.0 | 2.99 (2.21-3.77) |
|  | ≥70 | 101 | 17,328.9 | 5.83 (4.69-6.97) | 54 | 22,012.6 | 2.45 (1.80-3.11) |
| No DM | <40 | 4 | 5,766.8 | 0.69 (0.01-1.37) | 3 | 5,634.5 | 0.53 (0.00-1.13) |
|  | 40-49 | 6 | 13,892.1 | 0.43 (0.09-0.78) | 4 | 13,229.2 | 0.30 (0.01-0.6) |
|  | 50-59 | 34 | 28,183.9 | 1.21 (0.8-1.61) | 15 | 29,306.9 | 0.51 (0.25-0.77) |
|  | 60-69 | 36 | 11,342.0 | 3.17 (2.14-4.21) | 17 | 10,366.1 | 1.64 (0.86-2.42) |
|  | ≥70 | 51 | 13,285.3 | 3.84 (2.79-4.89) | 17 | 17,778.6 | 0.96 (0.50-1.41) |
