# Supplementary material for: Incidence rates of hepatocellular carcinoma based on risk stratification in steatotic liver disease for precision medicine: A real-world longitudinal nationwide study
Source: PLoS Med. 2024 Oct 25;21(10):e1004479. doi: 10.1371/journal.pmed.1004479 (PMC11548784; doi:10.1371/journal.pmed.1004479)
Supplement: S5 Table — (DOC) [file pmed.1004479.s007.doc]

S5 Table. HCC incidence rates per 1,000 person-years in subgroups of patients with SLD, stratified by a combination of non-liver cancer, sex, age, and DM

A Without non-liver cancer

| Patients |  | Male | | | Female | | |
| --- | --- | --- | --- | --- | --- | --- | --- |
|  | Age (years) | n | PY | Incidence rate per 1000 PY (95%CI) | n | PY | Incidence rate per 1000 PY (95%CI) |
| Total cohort | <40 | 9 | 225,763.4 | 0.04 (0.01-0.07) | 14 | 208,905.3 | 0.07 (0.03-0.10) |
|  | 40-49 | 30 | 305,410.3 | 0.10 (0.06-0.13) | 19 | 299,431.5 | 0.06 (0.03-0.09) |
|  | 50-59 | 126 | 338,025.3 | 0.37 (0.31-0.44) | 75 | 453,136.4 | 0.17 (0.13-0.20) |
|  | 60-69 | 127 | 89,290.2 | 1.42 (1.17-1.67) | 78 | 119,244.7 | 0.65 (0.51-0.80) |
|  | ≥70 | 70 | 31,274.9 | 2.24 (1.71-2.76) | 61 | 51,923.9 | 1.17 (0.88-1.47) |
| DM | <40 | 0 | 48,804.4 | 0.00 (0.00-0.00) | 6 | 58,010.9 | 0.10 (0.02-0.19) |
|  | 40-49 | 21 | 105,741.5 | 0.20 (0.11-0.28) | 11 | 120,734.5 | 0.09 (0.04-0.14) |
|  | 50-59 | 83 | 147,767.7 | 0.56 (0.44-0.68) | 55 | 203,570.1 | 0.27 (0.20-0.34) |
|  | 60-69 | 89 | 42,336.7 | 2.10 (1.67-2.54) | 59 | 55,655.3 | 1.06 (0.79-1.33) |
|  | ≥70 | 48 | 16,004.5 | 3.00 (2.15-3.85) | 37 | 25,167.7 | 1.47 (1.00-1.94) |
| No DM | <40 | 9 | 176,959.0 | 0.05 (0.02-0.08) | 8 | 150,894.4 | 0.05 (0.02-0.09) |
|  | 40-49 | 9 | 199,668.8 | 0.05 (0.02-0.07) | 8 | 178,697 | 0.04 (0.01-0.08) |
|  | 50-59 | 43 | 190,257.6 | 0.23 (0.16-0.29) | 20 | 249,566.4 | 0.08 (0.05-0.12) |
|  | 60-69 | 38 | 46,953.5 | 0.81 (0.55-1.07) | 19 | 63,589.4 | 0.30 (0.16-0.43) |
|  | ≥70 | 22 | 15,270.5 | 1.44 (0.84-2.04) | 24 | 26,756.1 | 0.90 (0.54-1.26) |

B With non-liver cancer

| Patients |  | Male | | | Female | | |
| --- | --- | --- | --- | --- | --- | --- | --- |
|  | Age (years) | n | PY | Incidence rate per 1000 PY (95%CI) | n | PY | Incidence rate per 1000 PY (95%CI) |
| Total cohort | <40 | 13 | 8,902.7 | 1.46 (0.67-2.25) | 16 | 11,297.0 | 1.42 (0.72-2.11) |
|  | 40-49 | 44 | 21,510.6 | 2.05 (1.44-2.65) | 39 | 36,596.8 | 1.07 (0.73-1.40) |
|  | 50-59 | 197 | 49,434.5 | 3.99 (3.43-4.54) | 137 | 79,509.0 | 1.72 (1.43-2.01) |
|  | 60-69 | 228 | 19,999.5 | 11.4 (9.92-12.88) | 137 | 26,257.9 | 5.22 (4.34-6.09) |
|  | ≥70 | 205 | 15,585.2 | 13.15 (11.35-14.95) | 105 | 18,667.1 | 5.62 (4.55-6.70) |
| DM | <40 | 5 | 2,376.3 | 2.10 (0.26-3.95) | 9 | 4,137.3 | 2.18 (0.75-3.60) |
|  | 40-49 | 22 | 8,134.0 | 2.70 (1.57-3.83) | 19 | 16,065.8 | 1.18 (0.65-1.71) |
|  | 50-59 | 128 | 22,874.3 | 5.60 (4.63-6.57) | 78 | 37,919.3 | 2.06 (1.60-2.51) |
|  | 60-69 | 168 | 10,052.4 | 16.71(14.19-19.24) | 91 | 13,396.4 | 6.79 (5.40-8.19) |
|  | ≥70 | 134 | 8,608.6 | 15.57 (12.93-18.20) | 73 | 10,040.3 | 7.27 (5.60-8.94) |
| No DM | <40 | 8 | 6,526.3 | 1.23 (0.38-2.08) | 7 | 7159.8 | 0.98 (0.25-1.70) |
|  | 40-49 | 22 | 13,376.6 | 1.64 (0.96-2.33) | 20 | 20531.0 | 0.97 (0.55-1.40) |
|  | 50-59 | 69 | 26,560.2 | 2.60 (1.98-3.21) | 59 | 41589.7 | 1.42 (1.06-1.78) |
|  | 60-69 | 60 | 9,947.1 | 6.03 (4.51-7.56) | 46 | 12861.6 | 3.58 (2.54-4.61) |
|  | ≥70 | 71 | 6,976.7 | 10.18 (7.81-12.54) | 32 | 8626.9 | 3.71 (2.42-4.99) |
