# Supplementary material for: Incidence rates of hepatocellular carcinoma based on risk stratification in steatotic liver disease for precision medicine: A real-world longitudinal nationwide study
Source: PLoS Med. 2024 Oct 25;21(10):e1004479. doi: 10.1371/journal.pmed.1004479 (PMC11548784; doi:10.1371/journal.pmed.1004479)
Supplement: S1 Fig — Note: *Other liver diseases: Autoimmune hepatitis, primary biliary cholangitis, primary sclerosing cholangitis, hereditary liver disease (alpha-1-antitrypsin deficiency, Wilson’s disease). ** HCC at baseline: any time before or at SLD diagnosis to 6 months after SLD diagnostic date. ICD, International Classification of Diseases; CM, Clinical Modification; SLD, Steatotic liver disease; HCC, hepatocellular carcinoma. (DOC) [file pmed.1004479.s008.doc]

S1 Figure. Patient Section from Merative Marketscan Research Databases

Note: *Other liver diseases: Autoimmune hepatitis, primary biliary cholangitis, primary sclerosing cholangitis, hereditary liver disease (alpha-1-antitrypsin deficiency, Wilson’s disease).

** HCC at baseline: any time before or at SLD diagnosis to 6 months after SLD diagnostic date.

Abbreviation: ICD, International Classification of Diseases; CM, Clinical Modification; SLD, Steatotic liver disease; HCC, hepatocellular carcinoma.
